# Child Maltreatment and Medical Traumatic Stress—A Double-Edged Sword

**DOI:** 10.3390/children12010017

**Published:** 2024-12-25

**Authors:** Rony Kapel Lev-ari, Roy Aloni, Amit Shalev, Avi Elbaz, Yael L. E. Ankri, Shiri Ben-David, Naomi Kahana Levy, Fortu Benarroch, Amichai Ben-Ari

**Affiliations:** 1 Department of Behavioral Sciences, Ariel University, Ariel 40700, Israel; roya@ariel.ac.il (R.A.); albeza@hadassah.org.il (A.E.); yael.errera@mail.huji.ac.il (Y.L.E.A.); amichai@hadassah.org.il (A.B.-A.); 2 New York State Psychiatric Institute and Department of Psychiatry, New York, NY 10032, USA; 3 Columbia University Vagelos College of Physicians & Surgeons, New York, NY 10032, USA; 4 Herman Dana Division of Child and Adolescent Psychiatry, Hadassah-Hebrew University Medical Center, Jerusalem 91240, Israel; amitshal@hadassah.org.il (A.S.); fortuben@hadassah.org.il (F.B.); 5 Department of Psychology, Ariel University, Ariel 40700, Israel; shirib@hadassah.org.il; 6 Department of Psychology, Hadassah-Hebrew University Medical Center, Jerusalem 91240, Israel; neomikl@tlvmc.gov.il; 7 Neurosurgery Department, Tel-Aviv Medical Center, Tel-Aviv 6801298, Israel

**Keywords:** medical traumatic stress, psychological distress, adverse childhood experiences, post-traumatic stress

## Abstract

Background/Objectives: Medical procedures can be a traumatic event for both children and their parents. Children who have experienced maltreatment or early traumatic experiences are at a higher risk for various emotional, behavioral, and health issues, including declining mental health. This may include experiencing heightened distress following medical procedures. The goal of this paper is to investigate the risk of distress symptoms following medical procedures for children with a history of child maltreatment vs. controls. Methods: A prospective study of 219 parents and children hospitalized in a pediatric surgical ward was conducted, with participants divided into study and control groups based on their reports of early traumatic experiences. Questionnaires measuring psychological distress were administered before the medical procedure and 3–5 months after discharge. Results: Children from the study group displayed significantly more distress symptoms before and after the procedure, with a substantial post-procedure increase. Parents of children who endured prior trauma and child maltreatment also exhibited elevated pre-procedure distress. Prior trauma and child maltreatment independently contributed to heightened medical distress. Post-procedure child distress was influenced by the early traumatic events and also by family support, socioeconomic status, and parental procedure-related post-traumatic stress symptoms. Conclusions: Children with a history of child maltreatment and trauma show an increased chance of psychological distress following medical procedures. Medical teams should be aware of this heightened risk and provide appropriate support.

## 1. Introduction

Enduring medical procedures is a stressful event for most children and parents [1]. Children suffering from serious illnesses or injuries sometimes undergo complex medical procedures that involve pain and even life-threatening conditions. Due to this, they often experience fear, uncertainty, and intense helplessness [2]. Studies have shown that painful or frightening medical procedures increase the likelihood of developing psychological distress and post-traumatic stress symptoms (PTSS) in children and their parents. For example, different studies found that between 10% and 30% of children hospitalized develop PTSS in the period between three and five months after hospitalization [3,4,5]. That said, because the manifestation of trauma in children is sometimes expressed in general psychological distress and not necessarily classic symptoms of trauma [6,7], it is essential to focus on the general mental state of children after medical procedures. Children show higher rates of general psychological distress following medical procedures, including a variety of internalizing and externalizing problems [8,9]. The high rate of distress among children after such medical procedures emphasizes the need for appropriate research and professional attention. Furthermore, it is important to broaden understanding of factors that influence children’s chances of showing signs of distress after medical intervention.

- **Risk Factors associated with general psychological distress following medical procedures:**

Studies in the field demonstrate a variety of factors that can impact the development of PTSS and general distress following a medical procedure. Factors impacting children’s distress following medical procedures can be grouped into different categories. These include factors that are associated with the procedure itself (e.g., the severity of the illness\injury\surgery; pain; more invasive procedures), factors associated with the parents’ prior or contemporary mental health, socioeconomic factors (e.g., poverty, minorities, residences in cultivation neighborhoods), features of the child (e.g., temperament, characteristics, anxiety levels), and finally other factors concerning the child (e.g., child maltreatment, prior abuse or neglect, prior traumatic events) (e.g., [4,10,11,12,13]). It seems that there is great value in identifying the children who are at higher risk and treating them before the onset of distress symptoms.

Children who have a history of childhood maltreatment are possibly at an even higher risk for serious mental health issues following medical procedures. Although there are a variety of studies regarding factors impacting mental health following medical procedures [3], only a few attempted to focus on children who endured maltreatment. This raises questions regarding these children’s vulnerability, especially due to their high chance of undergoing a medical procedure in the first place. It is also interesting to examine how these children are affected by additional risk factors that have been studied to affect children’s mental state after medical procedures.

- **Child maltreatment, early trauma, and mental health**

The World Health Organization (WHO) defines child maltreatment as all forms of violence against people under 18 years old, which results in actual or potential harm to the child’s health, development, or emotional and physical wellbeing. The WHO classified four forms of abuse, which include physical, emotional, sexual abuse, and neglect [14]. Most cases of reported maltreatment involve violence in the hands of the parents and other authority figures. Neglect is the most common form of child maltreatment [15]. All races, ethnicities, and socioeconomic groups can be affected by child maltreatment [16]. That said, it seems that child maltreatment is more likely to occur in households that also show household dysfunction, such as poverty, domestic violence at home, child disability, single-parent families, and parental mental health issues [17]. The effects of child maltreatment and early trauma have been extensively researched, with a variety of studies showing that they have a harmful effect on children’s physical and mental health (e.g., [18,19]).

Studies have consistently shown that child maltreatment leads to more mental health problems in children [20] and increases the child’s chance of suffering from mental health problems in adulthood (e.g., [19]). Those include internalizing and externalizing problems, suicidal ideation, post-traumatic stress disorder (PTSD), and complex trauma (e.g., [21,22,23]). Furthermore, child maltreatment and early trauma negatively impact children’s development, resulting in behavioral, emotional difficulties and difficulties regulating emotions (e.g., [16,24]). A recent study also showed that children who suffered from maltreatment in their childhood show lower levels of improvement when they receive treatment for mental health issues [25].

Children who have experienced different sorts of child maltreatment are more likely to be hospitalized and endure medical procedures as a direct or indirect result of maltreatment (e.g., [26]).

This current study aims to focus on the mental health of children following medical procedures. We will focus on different risk factors that impact the children’s state, with the focus being on the specific risk to children who have endured prior child maltreatment or trauma. Past traumas, especially early traumas, can impact the chance of the child developing PTSS after other extreme life events (e.g., [27]). Since these children are at high risk of developing chronic distress, the staff needs to have knowledge that will allow for them to identify these children and provide them with preventive care.

- **PTSS and general distress following medical procedures:**

According to the DSM-5 [28] exposure to life-threatening situations, such as medical procedures, can trigger post-traumatic stress disorder (PTSD). However, certain traumatic events can induce post-traumatic symptoms that fall short of the PTSD cutoff; these are referred to as PTSS [2,29], and manifest in anxiety, depression, avoidance, re-experiencing, and hyperarousal, potentially impacting a child’s recovery and long-term mental and physical health [30], often resulting in impaired functioning, sleep issues, emotional problems, and developmental challenges [4,5]. Studies regarding different medical procedures found that approximately 20–30% of children undergoing medical procedures showed PTSS (e.g., [5,10,31]).

Consequently, it is fundamental that medical staff are aware of risk and resilience factors in children who are about to undergo medical procedures in order to assist children who are at risk. Recent studies indicate that approximately 90% of healthcare professionals acknowledge the potential for mental distress linked to medical procedures and emphasize the necessity of addressing this during treatment [32]. Nonetheless, Bruce and colleagues’ research reveals that despite this heightened awareness, medical teams lack effective tools to identify and assist patients at risk of developing mental health issues. This study aims to enhance understanding of risk factors related to psychological distress post-medical procedures.

- **Double-edged sword—Child Maltreatment and Distress following medical procedures:**

As mentioned earlier, children with a history of child maltreatment are at risk for a variety of health and mental health problems (e.g., [23]). Primarily, these children are more likely to require medical treatment, some of which is directly related to the abuse they have experienced (e.g., [33,34]). Recent studies consistently show that children who endured maltreatment tend to have more medical interventions, suffer more from serious diseases, are more likely to have surgery, and have a higher chance of being hospitalized (e.g., [16,35]).

This leads to a double-edged sword, when children who are already exposed to trauma associated with the experiences they endured are more likely to acquire medical procedures that may lead to heightened psychological distress and mental health problems. That said, it remains a question whether these children are at a higher risk for showing distress symptoms after the procedure compared to children with no history of child maltreatment or trauma.

In recent years, several studies have attempted to gain a better understanding of PTSS and distress following medical procedures. In 2006, Kazak et al. devised the integrative model to grasp how children and families react psychologically during medical procedures. This framework comprises three sequential phases: The first encompasses events before the procedure, like accidents and diagnosis. The second involves the procedure itself, while the third focuses on the long-term post-medical traumatic stress (PMTS) experienced in the weeks or months afterward. The model also categorizes children seeking medical treatment into three risk levels. The “universal” category includes well-functioning families providing support without external help. The “targeted” level involves families facing challenges like poverty, needing psychosocial support. The “clinical” group involves families dealing with severe issues like substance abuse, requiring professional intervention to support the child.

This model deepens our understanding of high-risk children and families post-medical procedures. A recent meta-analysis confirmed and extended the integrative model’s assumptions. It was found that most families experience some distress symptoms following medical procedures, and while many recover, some may be left with symptoms that persist. Children with pre-existing mental health concerns, those from low socioeconomic backgrounds, and parents reacting strongly to the procedure face higher risks of long-lasting PMTS [36].

Although some studies have shown that prior mental health issues can impact distress following medical procedures, only a limited number of such studies have been conducted thus far (e.g., [10,37,38]). Furthermore, no study has specifically examined the risk of these symptoms in children with a history of maltreatment.

This study aims to enhance empirical and clinical knowledge by examining the relationship between childhood maltreatment and psychological distress post-medical procedures. It focuses on comparing distress levels between children at risk (specified as children with a history of some form of early trauma or child maltreatment) and a control group before and after a medical procedure. Additionally, it explores differences in the impact of other risk factors on distress between these groups.

The research hypotheses are as follows:

1. Distress levels will be significantly higher among children at risk after the medical procedure compared to those with no history of maltreatment.
2. Parents of children at risk will experience significantly higher distress related to their child’s medical procedure compared to control parents.
3. A moderation effect will reveal that children at risk respond differently to various risk factors (socioeconomic status, family support, procedure severity, parent’s pre-medical procedure distress) compared to controls.
4. A structural equation model (SEM) will illustrate associations between children at risk and controls, family support, parent’s distress, and socioeconomic state and their impact on distress 3–5 months after the medical procedure.

## 2. Materials and Methods

### 2.1. Participants

This study included 229 children hospitalized in the Pediatric Surgery Department at Hadassah-Hebrew University Medical Center. Participants were divided into two groups based on their responses to the TESI-PR questionnaire (see Section 2.3 for details on the division process). The study group comprised 39 children who reported experiencing adverse events or maltreatment prior to hospitalization, while the control group consisted of 190 children.

Although the study group was relatively small, we believe this division is crucial for understanding the unique needs of these children. Identifying such cases can be challenging, and it is possible that some instances of maltreatment were missed due to the inherent limitations of our assessment methods. Nevertheless, the study group, representing approximately 17% of the sample, aligns with the expected prevalence of these issues in similar populations [39,40].

Initial questioning occurred before procedures, involving various hospitalization reasons except head injuries. Surgical interventions spanned urology (26.7%), orthopedics (8.4%), ENT (11.3%), dermatology (11.3%), gastroenterology (8.4%), nephrology (8.4%), neurology (4.2%), cardiology (4.2%), combined (7%), and other (10.1%). Additionally, 46 participants (19.6%) were hospitalized without surgery. For 167 children (71.1%), it marked their inaugural surgical experience. Hospital stays ranged from 1 to 14 days, averaging 3.92 days (SD = 2.96). Control and study group children did not differ in the type of intervention and the number of times hospitalized.

The parents’ average age was 36.5, and children’s average age was 5.27. Children’s age ranged from 1 to 13, and we had 139 younger children (1–5) and 90 older children (6–13). Due to the wide age range, we ran an analysis showing that the younger and older children had no significant differences between them regarding the group that they were assigned to, CBCL before and after the surgery, and their demographics, including surgery severity, social economic background, parent’s prior mental health, parent’s education, and family support.

Among the children, 67% were boys and 33% were girls. Among subjects, 83% of parents were married, 7% were unmarried, and 10% did not report marital status. On the parents’ side, 60% were women and 40% were men. Parents voluntarily participated with consent, resulting in a sample of 39 study group participants and 180 controls. Group assignment details are provided in the study procedure.

### 2.2. Measures

In the present study, questionnaires were used to measure risk factors for the development of medical trauma, and questionnaires to measure the level of distress of the child and the parents in relation to the medical procedure. The questionnaires were all self-reported by the parents, with help and guidance by research assistants if necessary.

Achenbach’s Child Behavior Checklist (CBCL) is a parental self-report questionnaire assessing psychological and behavioral functions in children aged 1.5 to 18 [41]. The questionnaire includes eight scales (anxiety, depression, introversion, somatic complaints, attention problems, thinking problems, social problems, violation of rules, and aggressive behavior) with 100 items for 1.5- to 6-year-olds and 113 items for 6- to 18-year-olds. Responses range on a Likert scale of 0–2. This widely used tool has evaluated children’s emotional problems and behaviors in various studies (e.g., [42]). In our study, it assessed children’s mental state pre- and post-medical procedure. We employed the validated Hebrew version of the questionnaire, known for strong internal reliability (α = 0.74–0.89, [43]).

The Traumatic Events Screening Inventory—Parent Report (TESI-PR) is a parent-reported questionnaire gauging traumatic events in a child’s life. With 24 Yes/No questions, it covers experiences like neglect, abuse, and witnessed abuse (e.g., “Has your child ever been directly threatened?” or “Has your child witnessed family members physically harming each other?”). The questionnaire maintains strong internal reliability (α = 0.82, [44]).

The Posttraumatic Stress Diagnostic Scale (PDS-5) is a self-reported questionnaire for evaluating post-traumatic stress disorder (PTSD) symptoms in adults. It comprises 3 sub-scales, re-experiencing, arousal, and avoidance, with higher scores indicating greater PTSD symptom severity. We employed this questionnaire to assess PTSD symptoms in parents of hospitalized children. We utilized the validated Hebrew version [45] and obtained strong internal reliability of α = 0.97 in our study.

Psychosocial Assessment Tool 2.0 (PAT) is a self-report questionnaire, designed to assess psychosocial problems in children with life-threatening diseases and based on the Psychosocial and Prophylactic Health Model for Children (PPPHM) [46]. It contains various question types (yes/no, categorical, Likert scale) and comprises 84 items across 7 sub-scales: socioeconomic state, family social support, family problems, parent’s stress and mental state, family beliefs, child problems, and sibling problems. Higher scores indicate greater psychosocial risk. Each sub-scale’s items are scored dichotomously. In this study, we used the family social support, socioeconomic state, and parent’s stress and mental state. Internal consistency was fair at α = 0.70–0.81 [47].

### 2.3. Procedure

This study was approved by the Institutional Review Board of the medical center. Parents voluntarily participated in this study after being approached by our team. Those who agreed to participate signed an informed consent form and were asked to complete questionnaires. During this study, tests were conducted on two occasions: During the first, parents completed questionnaires near the time of the medical procedure, and during the second, which was conducted about three to five months after discharge from the hospital, the same parents were contacted via telephone and completed psychometric measures.

In the first phase of the trial, classification criteria were established for the study and control groups, based on the TESI-PR questionnaire. This questionnaire reviewed exposure to various types of traumatic events in the child’s life, including incidents of neglect and abuse. The researcher referred to parts of the questionnaire dealing with exposure to incidents of neglect and abuse according to the following categories: threat of severe bodily injury; severe violence between family members; threats of serious harm between family members; family member forcibly imprisoned; severe violence outside the family; sexual abuse; psychological abuse (e.g., severe threats of abandonment or expulsion from home); neglect (ongoing lack of fulfillment of basic needs, or care by a drug-addicted parent). Based on responses in the TESI-PR questionnaire, parents who reported their child experiencing any of the eight items were placed in the study group. This group included children who were deemed to have endured at least one event of child maltreatment. Those who did not report any of the eight items were categorized as the control group.

After defining the criteria, the researchers approached the parents of children treated in the surgical department in the hospital, with an offer to participate in this study. Those who agreed to participate received an explanation of the course of this study and were given an informed consent form that they signed. Following that, they filled out the questionnaires. The parents filled out the questionnaires in the presence of the researcher and under his guidance before the surgical procedure. As stated, 39 participants reported some form of child maltreatment or prior traumatic events and were classified as the study group. Table 1 demonstrates the prevalence of events among participants.

Of the participants in the study group, 19 met only one criterion, 13 met two criteria, 7 met three criteria, 6 met four criteria, 1 answered five criteria, and 1 met six criteria.

Aside from reporting prior maltreatment, the groups were found to be mostly homogenous, showing no significant differences between them in terms of age, religiosity, parental marriage status, education, socioeconomic status, family support factors, or severity of medical procedure. However, parents of children in the study group reported a significantly higher incidence of prior mental health issues compared to those in the control group.

In the second stage, three to five months after the medical procedure, the parents filled out a CBCL questionnaire to measure their child’s mental state post-medical procedure.

### 2.4. Data Analysis

Firstly, a missing data analysis was conducted, revealing a small percentage of missing values (2–8%). The data were found to be missing completely at random (MCAR) based on the results of Little’s MCAR test (χ2(57) = 57.442, *p* = 0.459). To handle the missing data, the multiple imputation method [48] was employed. Since the age range of our participants spanned from 1.5 to 18 years, parents completed separate surveys for two age groups: children aged 1 to 6 (n = 12) and children aged 6 to 18 (n = 38). As a result, certain analyses were conducted separately for each age group. To facilitate data integration and comparison across all age groups, standardized scores were applied to the CBCL questionnaire and the PTSD total scores. This standardized scoring method allowed for a unified score for each variable across the entire sample. It is important to mention that this standardized scoring approach has been widely used in previous studies [10]. To gain preliminary insights into the relationship between children’s mental state and parents’ mental state, Pearson correlations were employed. In the next step, we used logistic regression in order to further understand the factors impacting the likelihood of children showing clinical general distress. Subsequently, we conducted a MANOVA to examine differences in the children’s mental health based on their parents’ PTSD status (below vs. above cutoff). We obtained these using the cutoff for PTSD according to the PCL known criteria stating that scores above 33 indicate PTSD.

## 3. Results

### 3.1. The Differences Between Children with a History of Child Maltreatment and Controls Regarding Distress Levels Before and After the Medical Procedure

In order to test the hypothesis that the level of distress among the study group was significantly higher than in the control group, a repeated measures ANOVA was conducted. The dependent variable (DV) was the distress levels determined by the CBCL total scores. The independent variables were type of group (study or control) and measurement (before/after procedure). Details are shown in Table 2.

Multivariate results showed that the model as a whole was significant (Roy’s Largest Root = 0.55, F(1, 228) = 12.549, *p* < 0.001, ηp2 = 0.052). Furthermore, significant main effects were found regarding the group (F(1, 228) = 47.09, *p* < 0.001, ηp2 = 0.171) and the measurement (F(1, 228) = 20.41, *p* < 0.001, ηp2 = 0.082). Hence, overall, the study group showed more distress, and the levels of distress were significantly higher after the medical procedure.

**Figure 1:**
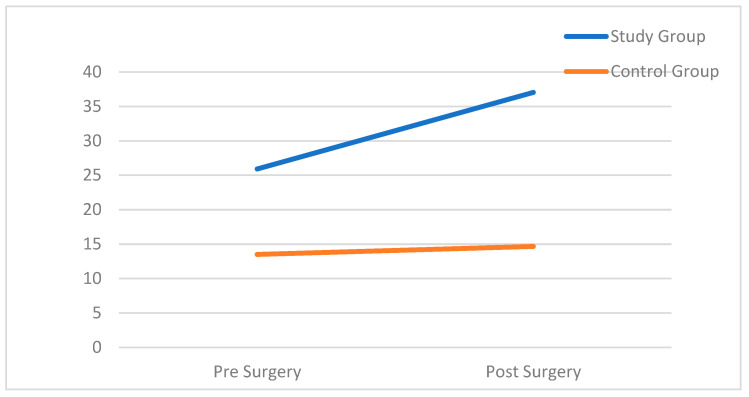
The change in distress before and after hospitalization.

In addition, an interaction effect was found (F(1, 228) = 12.54, *p* < 0.001, ηp2 = 0.052), showing that participants from the study group showed a greater increase in their distress levels compared to controls. A simple effects test with Bonferroni correction for multiple comparisons revealed that while among children in a control group the level of distress after hospitalization (M = 14.67) increased only slightly than before hospitalization (M = 13.51), among children at risk, the level of distress after hospitalization (M = 37.03) increased significantly compared to the level of distress before hospitalization (M = 25.92). Figure 1 demonstrates these differences.

### 3.2. Differences Between the Groups Regarding Other Risk Factors

We hypothesized that children at risk would differ from the control group regarding prior risk factors. We particularly suspected that parents of children in the study group would report greater levels of distress prior to hospitalization. To test this hypothesis, we conducted a MANOVA analysis, using the group (study/control) as the independent variable and a number of risk variables as the dependent variables (socioeconomic status; family support factors; severity of medical procedure; parent’s prior mental health; parent’s PTSD regarding the medical procedure). The findings indicated an overall significant main effect (Roy’s Largest Root = 0.134, F(1, 213) = 5.387, *p* < 0.001, ηp2 = 0.118). Further analysis showed no significant differences between the two groups concerning socioeconomic status (F(1, 217) = 0.183, *p* = n.s), family support factors (F(1, 217) = 0.330, *p* = n.s), or the severity of medical procedure (F(1, 217) = 0.025, *p* = n.s). However, the results revealed significant differences in the mental health status of the parents (F(1, 217) = 15.92, *p* < 0.001, ηp2 = 0.072), as well as the levels of PTSD symptoms the parents reported regarding the medical procedure (F(1, 217) = 9.07, *p* < 0.01, ηp2 = 0.042).

### 3.3. The Moderation Effect of Group (Study vs. Control) on Associations Between Different Factors and PMTS Symptoms

In order to examine the impact of children at risk, on the relationship between four selected additional risk factors and PMTS following the medical procedure, we conducted a regression analysis using PROCESS Model 1 [49]. In each model, the group was entered as a moderation variable, the dependent variable was the children’s CBCL levels after the procedure, and the independent variable was the specific risk factor (i.e., socioeconomic state, severity of medical procedure, support factors, parent’s mental state, and parent’s PTSD symptoms regarding the medical procedure). The Children’s CBCL levels before the procedure were added to all models as a covariant.

#### 3.3.1. Medical Procedure Severity

**Figure 2:**
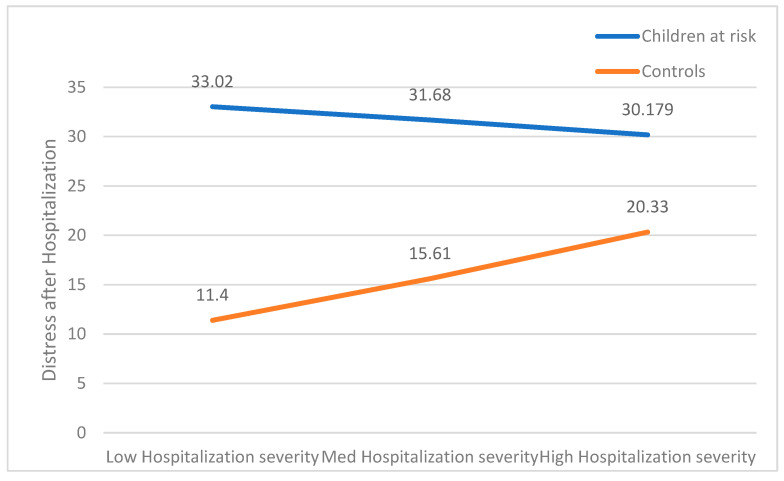
The association between PTSS and hospitalization severity moderated by group.

The overall model was statistically significant, accounting for 56.58% of the variation in CBCL levels (F(4, 225) = 73.28, *p* < 0.001). A Main effect was found regarding the group (b = −16.97, t(228) = −7.45, *p* < 0.001), but not regarding the medical procedure severity (b = −1.62, t(228) = −0.79, *p* = n.s). The interaction between the variables was significant as well (b = 6.71, t(223) = 3.01, *p* < 0.01). For a better understanding of the interaction found, a follow-up simple slopes analysis was conducted. The analysis showed a significant slope for the control group (b = 5.09, t(226) = 5.32, *p* < 0.001), but not for the study group (b = −1.62, t(226) = −0.787, *p* = n.s). These results are presented in Figure 2.

#### 3.3.2. Family Support Factors

**Figure 3:**
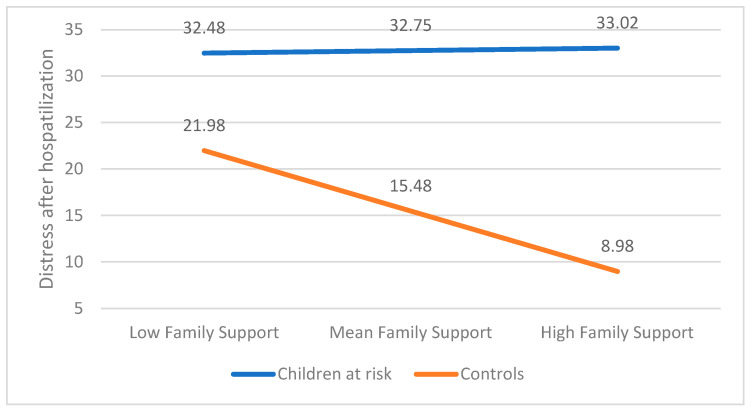
The association between distress after hospitalization and family support moderated by group.

The entire model was deemed significant and accounted for 59.41% of the variation in the CBCL levels following medical procedure (F(3, 228) = 82.35, *p* < 0.001). As before, a significant main effect was found regarding the group (b = −0.17.27, t(225) = −8.25, *p* < 0.001), but not regarding the family support factor (b = 0.06, t(225) = 0.157, *p* = n.s). However, the interaction between the two was found to be significant (b = −1.63, t(225) = −3.60, *p* < 0.001). Further analysis revealed that the study group did not exhibit any significant changes in the association between family support factors and distress following medical procedure (b = 0.063, t(225) = 0.157 *p* = n.s). However, a significant association was observed between support factors and distress outcome in the control group (b = −1.56, t(225) = −6.83, *p* < 0.001), indicating that family support factors had an impact on the distress outcome after medical procedure solely in the control group. These results are presented in Figure 3.

#### 3.3.3. Socioeconomic Factors

**Figure 4:**
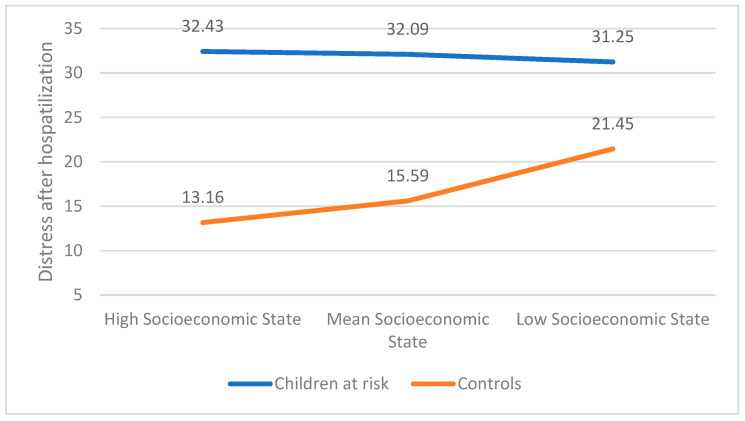
The association between distress after hospitalization and prior socioeconomic state moderated by group.

The overall model was statistically significant, accounting for 59.31% of the variation in CBCL levels (F(3, 228) = 81.63, *p* < 0.001). A significant main effect was found regarding the group (b = −0.16.50, t(225) = −7.89, *p* < 0.001), but not regarding the socioeconomic state (b = −1.59, t(225) = −0.446, *p* = n.s). As before, the interaction between the two was significant (b = 12.76, t(225) = 3.33, *p* < 0.01), with only the control group showing a significant change due to the socioeconomic state (b = 11.16, t(225) = 6.70, *p* < 0.001). Results are presented in Figure 4.

#### 3.3.4. Parental Mental Health State

The overall model was statistically significant, accounting for 64.77% of the variation in CBCL levels (F(3, 219) = 99.29, *p* < 0.001). Main effects were found for the group factor (b = −15.56, t(216) = −7.32, *p* < 0.001) and for the parental mental health variable (b = 0.363, t(216) = 2.03, *p* < 0.05). The interaction between them was significant as well (b = 0.414, t(216) = 2.23, *p* < 0.05). While both the study (b = 0.363, t(216) = 2.03, *p* < 0.05) and the control (b = 0.777, t(216) = 9.31, *p* < 0.001) groups showed significant slopes, the slope for the control group was more significant.

The association between child maltreatment, other risk factors, and distress following hospitalization:

To better understand the impact of child maltreatment on post-hospitalization distress, while accounting for other factors, we conducted a hierarchical regression analysis. The dependent variable was the post-hospitalization CBCL score. In the first step, we included factors that could influence distress (gender, age, socioeconomic status, family support, parental mental health, and hospitalization severity). In the second step, we added pre-hospitalization CBCL scores, and in the final step, we included the group variable (study vs. control). The results are presented in Table 3.

Taken together, the variables explained 65.4% of the variance in the children’s CBCL post-hospitalization. The results showed that adding the group variable yielded a significant F changes of 8.9% to the variance. Furthermore, the final model showed that age, parent’s distress, child’s pre-CBCL, and the group were significant predictors of distress post-hospitalization.

## 4. Discussion

The primary objective of this study was to gain a deeper understanding of the effects that medical procedures have on children who are at risk. Although a lot has been written about child maltreatment and its impact on children’s mental health (e.g., [20]) and about the effect of medical procedures on children (e.g., [10]), it seems there is a significant gap in the literature regarding the outcome of children at risk enduring medical procedures. The findings revealed that children who had experienced maltreatment were more susceptible to the effects of a medical procedure when compared to controls. Additionally, parents of these children were found to experience heightened levels of stress and distress regarding their child’s well-being. Furthermore, this study showed that other risk factors that affected the control group had less impact on these at-risk children. Finally, we conducted a regression analysis, demonstrating the prime factors that influence distress following hospitalization, while taking into consideration other known risk factors.

We hypothesized that children at risk will be more vulnerable to suffering from distress following a medical procedure compared to controls. Our findings align with previous research indicating that children with a history of maltreatment are more likely to display trauma symptoms following other stressful life events [50,51]. These results are concerning given that maltreated children are more susceptible to medical problems and injuries and are more likely to undergo medical procedures [52]. Furthermore, children at risk experience more stressful events, while the physiological consequences of stress in known to affect not only mental health but also non-psychological medical conditions (e.g., [53]). This double-edged sword increases their risk of experiencing mental health issues and medical problems. This results in a vicious cycle where prior child maltreatment leads to more medical procedures, which further increases the likelihood of developing mental health issues and distress concerning these procedures.

Several papers and clinical guidelines for medical staff focus on identifying children at risk of maltreatment when they arrive at medical facilities. These documents highlight factors that aid in the detection of abuse or neglect [54,55,56] and emphasize the importance of referring such children to welfare agencies (e.g., [57]). However, little has been written about the potential traumatic effects of medical procedures on these vulnerable children, which can add to their existing distress. Medical procedures can be perceived as life-threatening events, and children may experience physical pain, discomfort, and helplessness as a result (e.g., [13]), all of which can contribute to the development of psychological distress in all children, but in addition, children at risk often have lower levels of resilience, struggle to rely on their caregivers for support, and receive less support from their families and communities [23,58].

Our findings further support the idea that caregivers of at-risk children struggle to offer sufficient pre-procedure support. Parents in the study group exhibited greater trauma symptoms and mental health issues pre-procedure, indicating heightened distress. Existing research highlights strong links between parental distress before a medical procedure and a child’s post-procedure distress perception (e.g., [10,59]). Moreover, studies emphasize the impact of parental PTSD symptoms on the distress experienced by children during traumatic events (e.g., [60]). Furthermore, studies on at-risk children indicate that various factors contribute to the negative outcomes experienced by these children, including the proximity of caregivers who are dealing with additional mental health issues [61]. Our findings support this notion, revealing a significant difference in the psychological state of parents of at-risk children compared to those in the control group. As such, the high levels of distress and PTSD experienced by parents prior to a medical procedure represent a major risk factor that can impact both the child’s state before and after the procedure. These results emphasize the fact that these children are at excessive and complex risk. They suffer from the maltreatment itself and from their caregivers who are less able to support them, they tend to undergo more medical events to begin with, and the chances that these events will cause them greater distress is higher compared to children from the general population. As a result, it is very important to locate these children and families, considering the risks before starting the medical procedure, and referring them to further mental health treatment if necessary.

Our findings indicate that the study group did not exhibit greater sensitivity to risk factors compared to their counterparts in the control group. In fact, the results suggest that controls were more significantly impacted by variables such as the severity of the medical procedure, their socioeconomic status, and available support systems. These novel and intriguing findings might have different explanations. It is possible that prior child maltreatment may have a profound and overriding impact, overshadowing the influence of other factors. This is consistent with some studies, finding that resilience does not necessarily moderate the relationship between risk factors and distress (e.g., [62]) but contradicts others (e.g., [63]), additionally stating that some protective factors reduce the destructive impact of a history of child maltreatment. Further research is needed in order to obtain a clearer picture regarding the impact of additional risk and resilience factors on at-risk children undergoing medical procedures.

Our extended findings demonstrate that the factors that have the highest impact on distress following hospitalization were the child’s pre-hospitalization general distress, the parents’ levels of stress, and being in the study group. These results are in line with research in the field stating that a history of maltreatment impacts a child’s vulnerability when exposed to additional extreme life events [64] and in line with research stating that prior trauma can lead to higher levels of distress (e.g., [65]).

Past studies indicate that children from low-income households often exhibit trauma and distress after medical procedures [66]. Our model expands this insight by uncovering that children facing a history of maltreatment, regardless of family income, are at higher risk of trauma symptoms post-procedure. Similarly, parents’ traumatic reactions mirror this pattern. At-risk children tend to have parents with heightened trauma symptoms and, in turn, experience greater distress themselves. Our research bolsters this link [10,67] and establishes maltreatment history as a significant predictor of both parental distress post-procedure and the child’s emotional distress, even accounting for other factors. This suggests a parent–child distress cycle, possibly influencing PTSS in children. Importantly, parental distress impacts the child’s post-procedure distress, regardless of maltreatment presence, aligning with prior research emphasizing parent mental state impact [13,68].

### Limitations

This current study had serval limitations. Initially, we categorized our control and study groups using the TESI-PR questionnaire. This decision stemmed from our observation of children whose parents reported enduring adverse events, prompting our study’s focus on this area due to its underrepresented status in the existing literature. However, it is important to note that while this questionnaire provides a count of events experienced by the child, it lacks the capacity to gauge their severity and does not encompass all facets of childhood adverse events. For subsequent research, an alternative method could explore the influence of medical procedures on children with history of trauma and maltreatment, while offering a more comprehensive understanding that encompasses both the breadth and severity of the history of the events. Secondly, we had some cases of unevenness, with significantly more boys than girls participating in this study and our study group being much smaller than our control. This was due to our study method. Furthermore, our participants ranged in age from 1 to 13, and we acknowledge that distress may differ significantly between younger and older children. To address these issues, we conducted separate analyses, which showed no significant differences in distress or other factors between age groups or genders.

Furthermore, it is important to note that our sample size was relatively low (n = 229), with the study group being significantly smaller than the control group (n = 39). This was due to the prospective nature of this study. That said, this strengthens the need for future research in this topic, maybe conducting wider research with these populations.

Additionally, all questionnaires were based on self-reported or parental reports on their children. Although these are common methods in many studies [69], there are some limitations, especially due to the fact that we asked caregivers about their own behaviors toward the children, which might have biased their answers. Furthermore, children in our sample were young (m = 5.27), which led to us using the parents’ report in order to determine the children’s distress, which could have led to a less accurate understanding of the children’s state. That said, parental assessment of the child’s symptoms has been highly correlated with their children’s self-reports [70]. Future research can rely on other more unbiased methods to determine the children’s distress levels.

## 5. Conclusions

This study has important clinical implications, especially for children with a history of child maltreatment. Previous research has shown that these children are at higher risk of psychological and health issues throughout their lives, including increased medical problems. However, there is limited research on their mental health risk. Moreover, they are more susceptible to distress symptoms and medical procedures. Currently, hospitals lack interventions to address the mental health needs of these families alongside medical treatment.

This study reveals that factors promoting resilience in other children have less impact on those at risk. Parents of these children also experience more distress during medical procedures, leading to a cycle of distress that affects the child’s recovery.

However, medical staff can be trained to detect and support these families, potentially preventing the double vulnerability of medical procedures and early traumas. Medical settings offer a crucial opportunity to identify and support these vulnerable children, facilitating access to professional mental health care before, during, and after medical procedures. By doing so, the risk of post-procedure mental health issues may be reduced. Medical staff can also educate these families about their heightened risk and provide coping tools for trauma symptoms related to the medical procedure.

In conclusion, identifying and supporting children at risk during medical procedures is vital for improving their long-term outcomes.

## Figures and Tables

**Table 1:** Prevalence of participants for each item of neglect and abuse.

Threat of Severe Sabotage	Domestic Violence	Threats in the Family	Family Member Incarceration	Violence Outside the Family	Sexual Abuse	Psychological Abuse	Neglect
16	21	37	12	11	0	16	1

**Table 2:** Average scores, standard deviations, and group differences in the pre- and postoperative CBCL questionnaire.

	Study Group		Control Group		Total	
	M	SD	M	SD	M	SD
CBCL pre-medical procedure	25.92	17.79	13.51	18.04	15.72	18.58
CBCL post-medical procedure	37.03	15.44	14.66	15.98	18.65	18.02
Total	31.47	16.61	14.08	17.01	17.18	18.3

**Table 3:** Hierarchical regression: predictors of CBCL rates post-surgery.

Variables				
	B	Std Error	β	R^2^ Change
Step 1:				52% ***
Gender	1.89	1.82	0.050	
Age	0.524	0.243	0.108 *	
Socioeconomic state	0.383	2.102	0.11	
Surgery severity	0.300	1.056	0.016	
Family support	−0.308	0.268	−0.072	
Parent’s prior mental health	−1.625	0.647	−0.168 *	
Parent’s distress	0.836	0.114	0.532 ***	
Step 2:				6.0% ***
Gender	2.16	1.76	0.057	
Age	0.536	0.228	0.110 *	
Socioeconomic state	1.36	1.97	0.40	
Surgery severity	0.182	0.991	0.09	
Family support	−0.118	0.254	−0.027	
Parent’s prior mental health	−0.630	0.633	−0.065	
Parent’s distress	0.671	0.426	0.426 ***	
CBCL—pre-surgery	0.308	0.319	0.329 ***	
Step 3:				8.9% ***
Gender	1.04	1.53	0.028	
Age	0.477	0.203	0.098 *	
Socioeconomic state	2.48	1.768	0.074	
Surgery severity	0.854	0.887	0.044	
Family support	−0.347	0.228	−0.081	
Parent’s prior mental health	−0.568	0.564	−0.059	
Parent’s distress	0.536	0.100	0.341 ***	
CBCL—pre-surgery	0.235	0.051	0.244 ***	
Group (study vs. control)	−14.862	1.988	−0.320 ***	
R^2^ Total	65.4% ***			

* *p* < 0.05; *** *p* < 0.001.

## Data Availability

The data presented in this study are available on request from the corresponding author due to privacy concerns.
